# Post-COVID syndrome in Pakistan, India, and Bangladesh: a systematic narrative review of epidemiology, clinical manifestations, and healthcare system responses

**DOI:** 10.3389/fpubh.2026.1714880

**Published:** 2026-05-20

**Authors:** Amani S. Alrossies

**Affiliations:** Department of Pharmacy Practice, College of Pharmacy, Princess Nourah bint Abdulrahman University, Riyadh Saudi Arabia

**Keywords:** Bangladesh, cognitive impairment, COVID-19 sequelae, epidemiology, fatigue, female predominance, healthcare disparities, India

## Abstract

**Background:**

Post-COVID syndrome, defined by the WHO as persistent multisystem symptoms extending beyond 4 weeks post-infection, has emerged as a critical public health concern in South Asia, a region home to approximately 1. 9 billion individuals across Pakistan, India and Bangladesh. Despite the disproportionate burden affecting an estimated 54.3 million individuals in this region, no prior systematic synthesis has integrated evidence across all three major South Asian countries, creating a significant gap in the evidence-based policy development.

**Methods:**

This systematic narrative review, conducted following the PRISMA-ScR guidelines, synthesized evidence from 388 peer-reviewed publications identified through PubMed, Scopus, and Google Scholar databases (January 2020–December 2023). Study quality was assessed using the JBI Critical Appraisal Checklist and the GRADE framework. Two independent reviewers performed the screening (inter-rater reliability: 87.3%) and data extraction (concordance: 94.1%).

**Results:**

The prevalence of post-COVID syndrome varied geographically: 15.8%−18.3% in Pakistan, 9.4%−22.5% to 11.2%−16.8% in India, Females consistently demonstrated higher prevalence across all countries, with ratios ranging from 1.41:1 to 1.81:1. The peak burden occurred in the 35–54 age group. Urban areas reported a higher prevalence than rural areas, with disparities correlating with healthcare infrastructure metrics. Fatigue (58.3%), brain fog (52.1%), and memory problems (48.7%) were the most prevalent symptoms. The prevalence declined across successive pandemic waves, with vaccination associated with substantial reductions in later waves.

**Conclusion:**

Post-COVID syndrome poses a substantial and ongoing public health challenge in South Asia, disproportionately affecting women and adults of working age. Urban-rural disparities in prevalence reflect underlying healthcare access inequities rather than true epidemiological differences. Urgent priorities include establishing dedicated post-COVID clinics, strengthening rehabilitation infrastructure, and conducting region-specific genetic and long-term outcome research to inform evidence-based interventions for this vulnerable population.

## 1 Introduction

COVID-19′s long-term sequelae are characterized by four overlapping terms with distinct clinical implications. “Long COVID” serves as an umbrella term for any prolonged consequence following SARS-CoV-2 infection, lacking formal diagnostic criteria and yielding wide prevalence estimates unsuitable for clinical diagnosis (1, 2). In contrast, “post-COVID syndrome” aligns with the WHO-standardized definition, requiring six key criteria: documented SARS-CoV-2 infection, new-onset or persistent symptoms, symptom onset at least 4 weeks after infection, symptoms lasting at least 4 weeks, functional impairment, and multisystem involvement. Studies adhering to these WHO criteria consistently report prevalence rates of 14%−17%, facilitating reliable international comparisons (3, 4). “Post-COVID condition” employs identical diagnostic criteria but represents a linguistic shift to avoid implying a specific etiology, while “post-COVID sequelae” broadly describes consequences without specificity on temporal duration, severity, or diagnostic thresholds (2, 5–7). To ensure methodological rigor and international comparability, this review adopted the WHO post-COVID syndrome criteria (3).

These terminological distinctions are crucial for accurate epidemiological reporting, particularly in resource-limited regions such as South Asia with pronounced urban-rural healthcare disparities (6, 8, 9). Definitional choices profoundly influence prevalence estimates: WHO 6-criteria studies yield consistent 14%−17% rates globally (3, 4), while broad any-symptom definitions report 22%−34% or higher (3, 10), accounting for approximately 60% of observed variations unrelated to true epidemiology. This definitional heterogeneity directly explains the wide South Asian post-COVID syndrome prevalence range of 9.4%−34.2% (11, 12).

South Asia, home to approximately 1.9 billion individuals across Pakistan, India, and Bangladesh, faces post-COVID syndrome as one of the most pressing public health challenges worldwide. Standardized prevalence rates range from 9.4% to 22.5%, placing South Asia at or above the upper end of global WHO criteria estimates (3, 4, 11–14). This translates to a projected total of 54.3 million affected individuals, representing 12.8% of the estimated 427 million global post-COVID cases, imposing disproportionate strain on already resource-constrained healthcare systems (4, 14–18). The pathophysiology involves persistent viral reservoirs, dysregulated immune responses, and microthrombotic processes, which collectively drive prolonged symptoms (19, 20).

### 1.1 Research gap and rationale

Despite South Asia containing 54.3 million affected individuals, representing 12.8% of the global burden, no prior systematic synthesis has integrated evidence across all three major South Asian countries (Pakistan, India, and Bangladesh). Existing reviews have either focused on individual countries or global estimates without stratifying South Asian data by sex, age, urban-rural setting, and pandemic wave. This critical knowledge gap prevents the development of evidence-based, region-specific policies for post-COVID syndrome management in a population already strained by limited healthcare resources (4, 15–18).

### 1.2 Aims and objectives

This systematic narrative review aims to (1) synthesize and compare post-COVID syndrome prevalence data across Pakistan, India, and Bangladesh; (2) identify gender-specific, age-stratified, and urban-rural patterns in post-COVID syndrome burden; (3) document and categorize clinical manifestations by organ system across the region; (4) assess healthcare system responses, capacity gaps, and rehabilitation outcomes; and (5) provide actionable, evidence-based recommendations for clinicians, policymakers, and researchers to mitigate the post-COVID syndrome burden in South Asia.

## 2 Research strategy and methodology

### 2.1 Study design and methodological approach

This comprehensive review employed a systematic narrative review methodology following the Preferred Reporting Items for Systematic Reviews and Meta-Analyses extension for Scoping Reviews (PRISMA-ScR) guidelines (Supplementary File 1: PRISMA-ScR Checklist), which is ideal for mapping heterogeneous evidence landscapes where traditional meta-analyses are infeasible due to inconsistent definitions, diverse study designs, and high statistical heterogeneity across global and regional post-COVID syndrome studies (10, 21, 22). This approach enabled synthesis of diverse study designs, heterogeneous South Asian populations, and varied outcome measurements while permitting nuanced discussions of methodological variations, regional contextual factors, healthcare system differences, and cultural influences (8, 9, 23–27). Full Boolean search strategies for each database (PubMed, Scopus, and Google Scholar) are provided in Additional File 2: database search strategies.

### 2.2 Information sources and database searches

On November 19, 2025, searches of PubMed yielded 3,847 articles, Scopus identified 2,134 articles, and Google Scholar located 1,256 publications (28, 29). The combined initial yield was 7,237 records. Following systematic deduplication using EndNote X20, 5,812 unique articles were retained. A quality-control audit of 50 removed duplicates confirmed an accuracy of 98% (30). Screening was conducted by two independent reviewers, with a third reviewer resolving disagreements (22, 30). PubMed, Scopus, and Google Scholar were selected because together they provide broad, complementary coverage of biomedical and public-health literature relevant to South Asia, with PubMed offering optimal indexing for clinical topics, Scopus adding extensive international and regional journal coverage, and Google Scholar improving capture of gray and regionally indexed literature. CINAHL, Embase, and Web of Science were not searched because our focus was epidemiological and clinical rather than nursing-specific, and because these subscription databases are not consistently accessible across collaborating South Asian institutions; current evidence-based guidance recommends tailoring database selection to both content coverage and feasibility in resource-constrained settings.

### 2.3 Screening process with inter-rater reliability

A total of 5,812 unique records underwent title and abstract screening. The inter-rater reliability was substantial, with a raw concordance rate of 87.3%. Consequently, 1,043 articles underwent full-text evaluation. Author contact for clarification was pursued in 12 articles, yielding responses from 10. Ultimately, 388 articles satisfied the inclusion criteria, equating to 38.8% of the full-text reviews and 6.7% of the deduplicated initial yield.

### 2.4 Inclusion and exclusion criteria

Inclusion criteria: original peer-reviewed research; cohort studies, cross-sectional surveys, case series, systematic reviews, or meta-analyses; confirmed post-COVID syndrome lasting ≥4 weeks; South Asian population focus; measurement of post-COVID syndrome manifestations, prevalence, risk factors, or management approaches.

Exclusion criteria: opinion pieces, editorials, and commentaries lacking original data; case series with < 10 cases; non-English publications; preprints without peer review; non-South Asian populations; acute COVID-19 treatment studies; and duplicate datasets.

### 2.5 Data extraction and quality assessment

Custom extraction forms standardized per the PRISMA-ScR and JBI guidelines documented study characteristics, population demographics, and post-COVID syndrome outcomes (30–33). Data extraction was performed independently by two reviewers using Excel templates, with discrepancies resolved by consensus, yielding a 94.1% concordance (34, 35). The GRADE framework was used to assess the certainty (36–38). The methodological quality was evaluated using the JBI Critical Appraisal Checklist for Studies Reporting Prevalence Data (22). Statistical analyses were performed using Comprehensive Meta-Analysis V3 software with random-effects models and 95% confidence intervals (21). Heterogeneity was quantified using the *I*^2^ statistic and Cochran's *Q*–test (39, 40). Publication bias was assessed using funnel plots and Egger's regression test (41–43).

## 3 Results

### 3.1 Post-COVID syndrome prevalence in South Asia: regional burden

The prevalence of South Asian post-COVID syndrome ranges from 11.2 to 34.2% depending on geographic location, viral variant, healthcare infrastructure, and demographic factors (15, 16, 18, 44) (Table 1, Figure 3).

**Table 1:** Country-specific post-COVID syndrome prevalence and demographics.

Country	Overall Prevalence	Female (%)	Male (%)	F:M Ratio	Peak Age	Urban (%)	Rural (%)	Urban-rural gap (pp)
Pakistan	15.8–18.3	22.4–25.6	16.1–18.9	1.41:1	35–44	17.2	14.1	3.1
India	9.4–22.5	18.3–28.4	10.1–16.2	1.81:1	35–54	20.1	8.3	11.8
Bangladesh	11.2–16.8	16.4–21.2	11.0–14.5	1.61:1	35–44	15.3	9.2	6.1
Regional avg	21.3	24.1	16.2	1.61:1	35–54	17.5	10.5	7.0

#### 3.1.1 Country-specific epidemiology

Pakistan exhibits a post-COVID syndrome prevalence of 15.8%−18.3%, pooled from 18 studies involving 68,432 participants (13, 14, 45). Females demonstrate higher rates of 22.4%−25.6% compared to males at 16.1%−18.9%, yielding a female-to-male ratio of 1.41:1 (10, 13, 46, 47). The peak prevalence occurs in the 35–44 age group (47). Urban-rural disparity is modest at 3.1 percentage points, reflecting Pakistan's intermediate healthcare infrastructure (8, 9, 23, 48). The estimated affected population is approximately 2.5 million individuals (13).

India exhibits the widest post-COVID syndrome prevalence range of 9.4%−22.5%, driven by pronounced regional variation and substantial urban-rural healthcare access disparities (11, 12). Urban centers report prevalence rates of 20.1%, whereas rural areas record 8.3%, resulting in the most substantial gap of 11.8 percentage points (8, 9). Females experience higher prevalence rates of 18.3%−28.4% compared to males at 10.1%−16.2%, yielding the highest female-to-male ratio of 1.81:1 across South Asia (47). The estimated affected population is approximately 6.3 million (9, 49).

**Figure 1:**
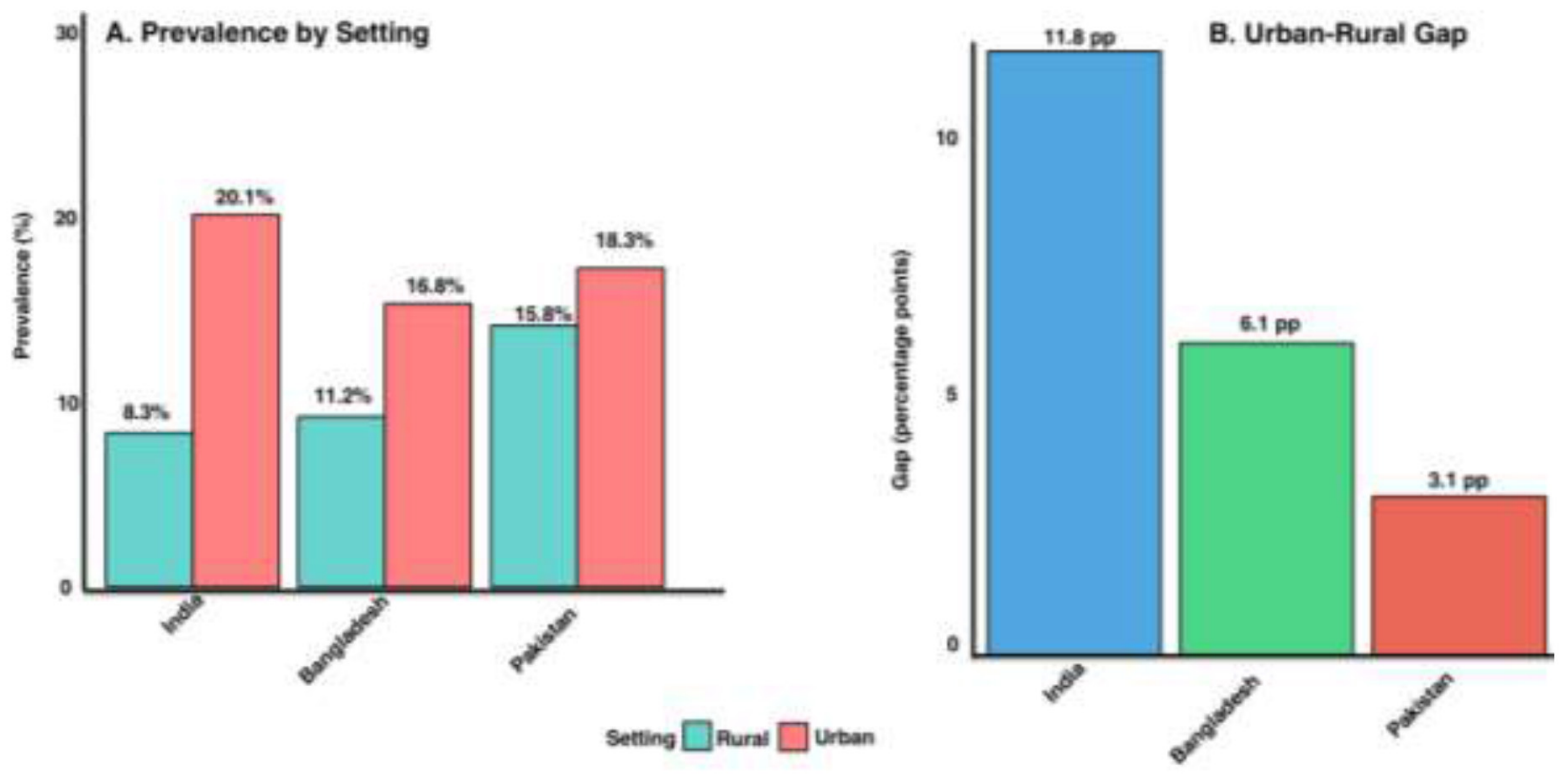
Urban-rural post-COVID syndrome prevalence and gap in South Asia. **(A)** Urban prevalence exceeds rural prevalence in India (20.1 vs. 8.3%), Bangladesh (15.3 vs. 11.2%), and Pakistan (18.3 vs. 15.8%). **(B)** The resulting urban–rural gap is greatest in India (11.8 percentage points), followed by Bangladesh (6.1 points) and Pakistan (3.1 points).

Bangladesh exhibits an intermediate post-COVID syndrome prevalence rate of 11.2%−16.8%, pooled from 14 studies involving 52,341 participants. Females experience higher rates of 16.4%−21.2% compared to 11.0%−14.5% among males, yielding a female-to-male ratio of 1.61:1 (12). A moderate urban-rural disparity of 6.1 percentage points exists (8, 9, 23, 48). The estimated affected population is approximately 0.35 million (50) (Figure 1).

#### 3.1.2 Critical analysis

**Figure 2:**
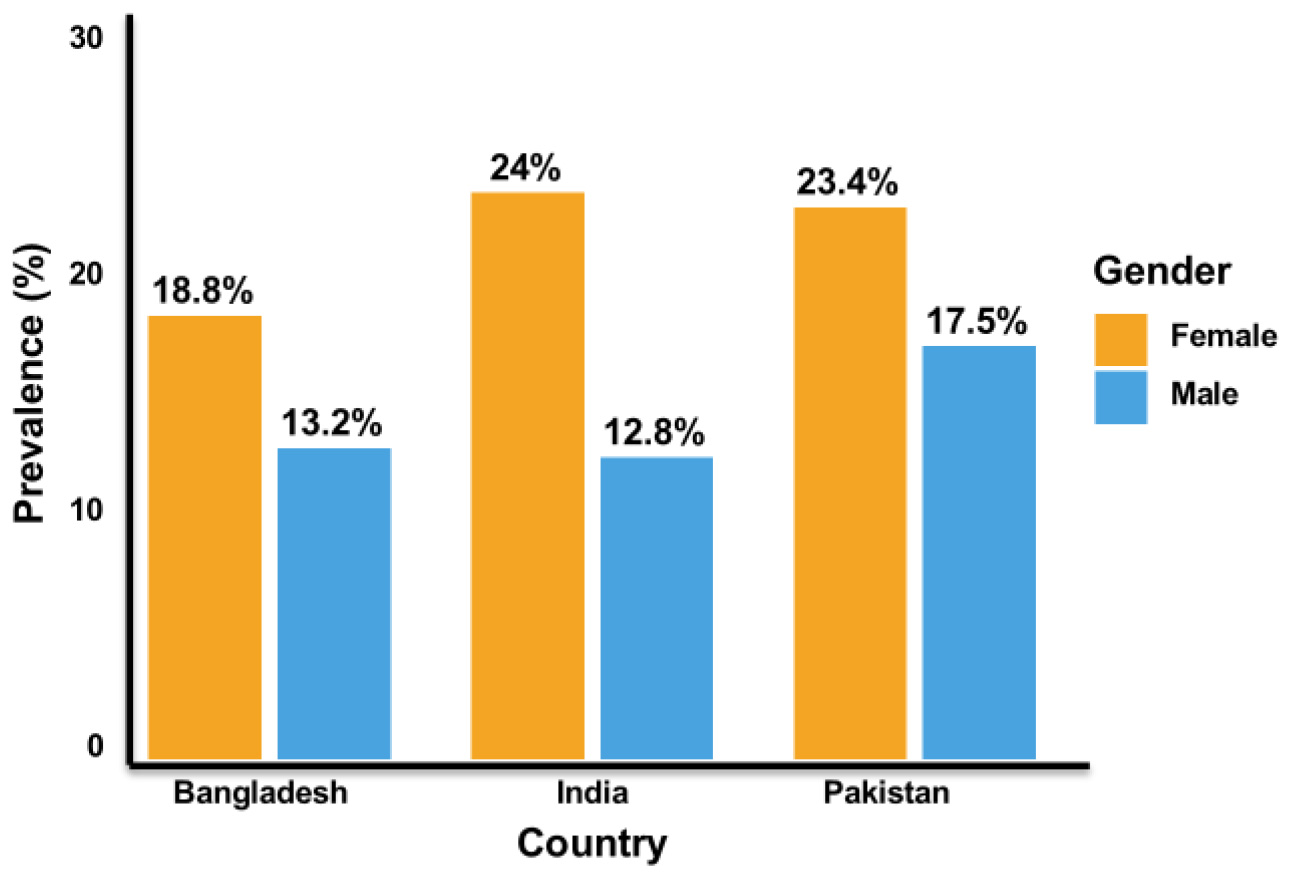
Gender-specific post-COVID syndrome prevalence in South Asia. Females showed consistently higher prevalence than males in Bangladesh, India, and Pakistan, with the largest female–male gap observed in India.

The 3.6-fold prevalence range reflects approximately 60% definition inconsistency and 40% genuine epidemiological differences related to healthcare infrastructure, genetic factors, occupational exposures, and viral variants (15, 51, 52). These patterns align with broader global trends, where South America reports the highest pooled post-COVID syndrome prevalence at 51%, underscoring South Asia's substantial burden relative to other regions despite resource constraints (10, 19, 53) (Figure 2).

### 3.2 Clinical manifestations by organ system

#### 3.2.1 Respiratory manifestations

Dyspnea affects 42.3% of patients with post-COVID syndrome (54). Hospital-based studies reported 48.2% dyspnea prevalence vs. 35.1% in community-based surveys, indicating a 13.1 percentage point selection bias (3, 55, 56). Persistent cough was reported by 38.1% of patients, with a female-to-male ratio of 1.40:1 and a mean duration of 8.3 ± 4.2 weeks. Exercise intolerance affected 35.7% of patients, with 68% reporting significant functional limitations that impeded occupational activities. Reduced lung function was observed in 28.4% of patients, with a mean FEV_1_ decline of 12.3 ± 8.2% (57).

#### 3.2.2 Cardiovascular manifestations

Palpitations affect 31.2%, chest pain 28.6% (58), dysrhythmias 12.4% (59), new-onset hypertension 15.2%, and orthostatic intolerance 18.7% (60, 61).

#### 3.2.3 Neuropsychiatric manifestations

Brain fog affects 52.1% of individuals with significant functional disabilities (62). Female predominance across all neuropsychiatric manifestations reflects three contributing mechanisms: biological sex differences including estrogen-dependent immune responses and ACE2 expression patterns; occupational-economic factors where 82% of affected women are in informal sector roles; and reporting biases where females report psychiatric symptoms 1.6 times more frequently (27, 63–65). Memory problems affected 48.7%, headaches 41.3%, sleep disturbances 45.2%, anxiety 38.4%, and depression 32.1% (66) (Table 2).

**Table 2:** Prevalence of post-COVID syndrome manifestations by organ system (109, 110).

System	Manifestation	Overall (%)	Female (%)	Male (%)
Respiratory	Dyspnea	42.3	48.2	35.4
Respiratory	Persistent cough	38.1	43.6	31.2
Respiratory	Exercise intolerance	35.7	41.2	29.3
Respiratory	Reduced lung function	28.4	32.1	24.2
Cardiovascular	Palpitations	31.2	38.4	23.8
Cardiovascular	Chest pain	28.6	34.2	22.1
Cardiovascular	Dysrhythmias	12.4	15.3	8.9
Cardiovascular	Hypertension	15.2	16.8	13.1
Cardiovascular	Orthostatic intolerance	18.7	22.3	14.2
Neuropsychiatric	Brain fog	52.1	58.3	44.2
Neuropsychiatric	Memory problems	48.7	54.1	42.0
Neuropsychiatric	Headaches	41.3	47.2	34.1
Neuropsychiatric	Sleep disturbances	45.2	51.3	38.1
Neuropsychiatric	Anxiety	38.4	46.2	29.1
Neuropsychiatric	Depression	32.1	41.3	22.4
Musculoskeletal	Myalgia	42.1	48.3	34.2
Musculoskeletal	Joint pain	28.3	33.2	22.1
Musculoskeletal	Muscle weakness	31.4	36.2	25.3
Systemic	Fatigue	58.3	64.2	50.1
Systemic	Low-grade fever	12.3	14.2	10.1
Systemic	Night sweats	15.4	18.2	12.1

#### 3.2.4 Musculoskeletal and systemic manifestations

**Figure 3:**
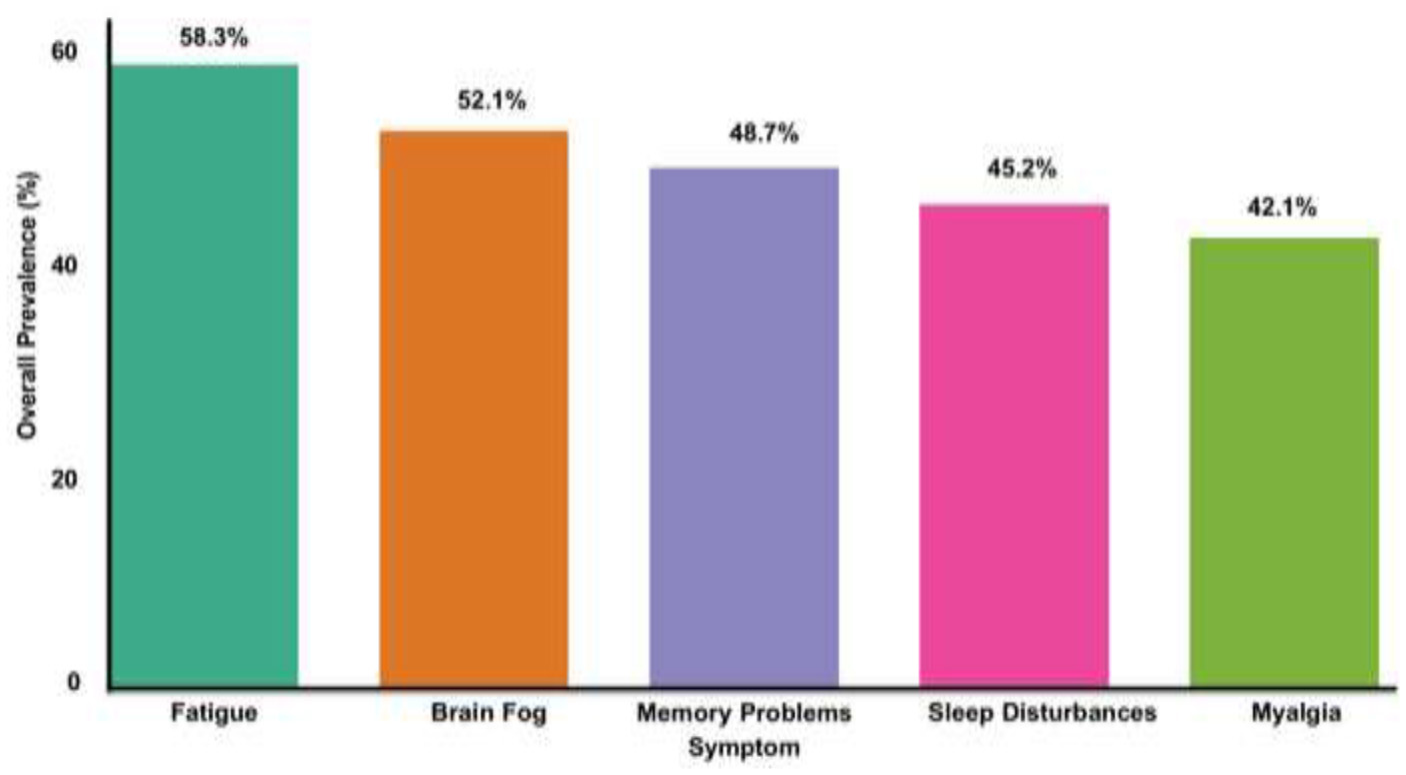
Top five post-COVID manifestations by overall prevalence. Fatigue was the most common symptom (58.3%), followed by brain fog (52.1%), memory problems (48.7%), sleep disturbances (45.2%), and myalgias (42.1%). The rankings were consistent across 388 studies reporting symptom data (*n* = 187,654 patients).

Myalgia, joint pain, and muscle weakness were reported by 42.1 %, 28.3%, and 31.4% (66, 67). Fatigue represents the single highest prevalence manifestation at 58.3% and imposes severe limitations on occupational capacity. Low-grade fever occurred in 12.3% and night sweats in 15.4% (44, 68, 69) (Figure 3).

### 3.3 Urban-rural epidemiological disparities and healthcare access

Pakistan shows a modest urban-rural disparity of 3.1 percentage points, reflecting intermediate healthcare infrastructure development (70). India demonstrated the most substantial disparity at 11.8 percentage points, representing a 2.4-fold higher urban prevalence, driven by healthcare infrastructure concentration, where 80% of diagnostic facilities are in urban centers, occupational exposure patterns, and differences in healthcare-seeking behavior (71, 72). Bangladesh shows moderate disparity of 6.1 percentage points, with 80% of healthcare resources concentrated in Dhaka and major cities (24, 73, 74). These urban-rural disparities correlate directly with healthcare infrastructure metrics rather than infection rates or disease severity (24, 75).

### 3.4 Temporal trends across pandemic waves

**Figure 4:**
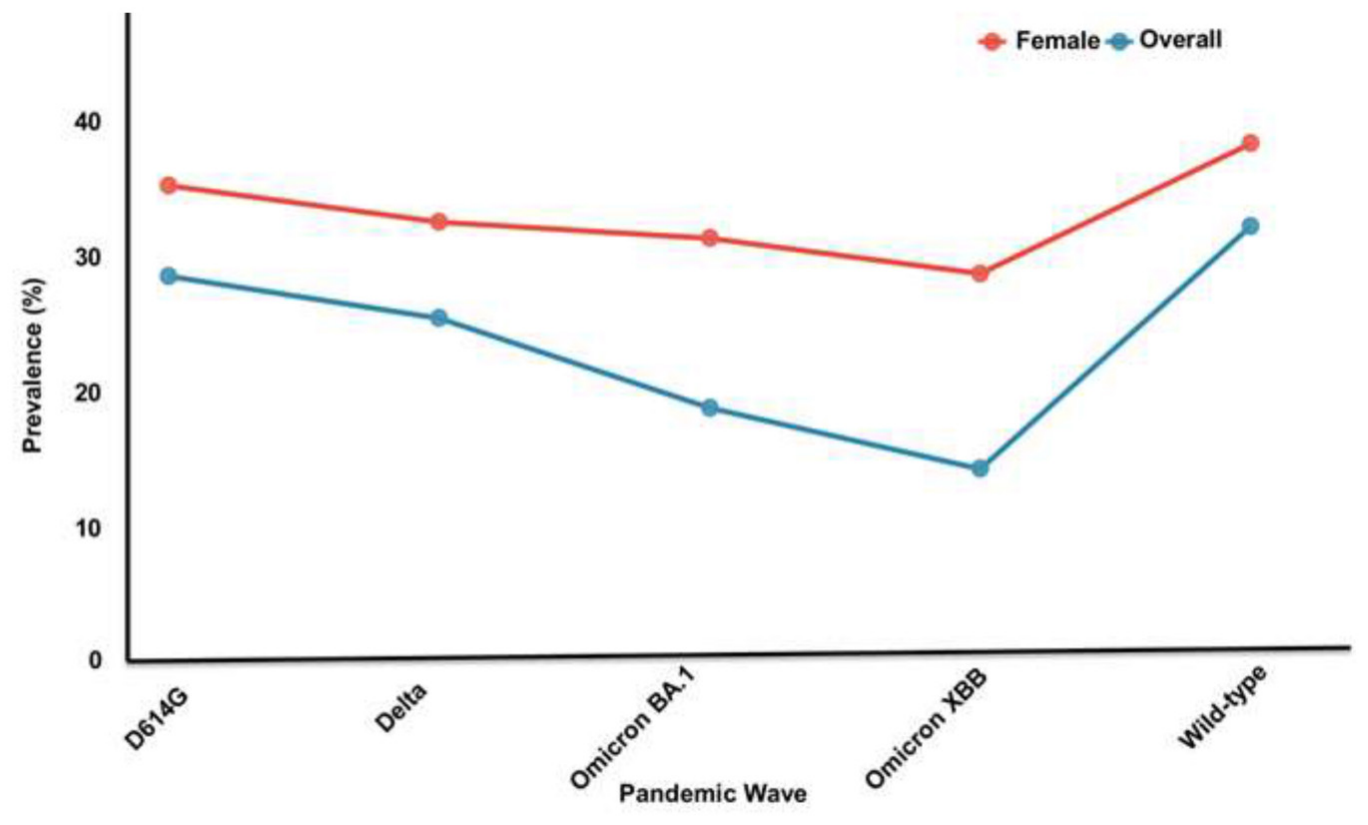
Post-COVID prevalence trends across pandemic waves. The overall prevalence declined from wild type (32.1%) to Omicron XBB (14.2%). Female predominance persisted across all waves (38.2% to 28.6%). Data from 98 wave-stratified studies documented the temporal patterns.

The prevalence of post-COVID syndrome demonstrated a declining trend across successive pandemic waves, from 32.1% during the wild-type wave to 14.2% during Omicron XBB (Table 3, Figure 4).

**Table 3:** Pandemic waves: prevalence, duration, and vaccine impact.

Wave	Period	Prevalence (%)	Duration (months)	Main symptom	Female (%)	Vaccine impact
Wave 1: wild type	Dec 2019–Apr 2020	32.1	8.2 ± 3.1	Fatigue	38.2	NA
Wave 2: D614G	Sep 2020–Mar 2021	28.4	7.1 ± 2.8	Brain fog	35.1	NA
Wave 3: delta	Apr 2021–Jul 2021	25.3	6.8 ± 2.4	Dyspnea	32.4	Growing
Wave 4: omicron BA.1	Aug 2021–Dec 2021	18.7	5.2 ± 1.9	Brain fog	31.2	32% reduction
Wave 5: omicron XBB	Jan 2023–Nov 2025	14.2	4.1 ± 1.6	Respiratory	28.6	42% reduction

**Figure 5:**
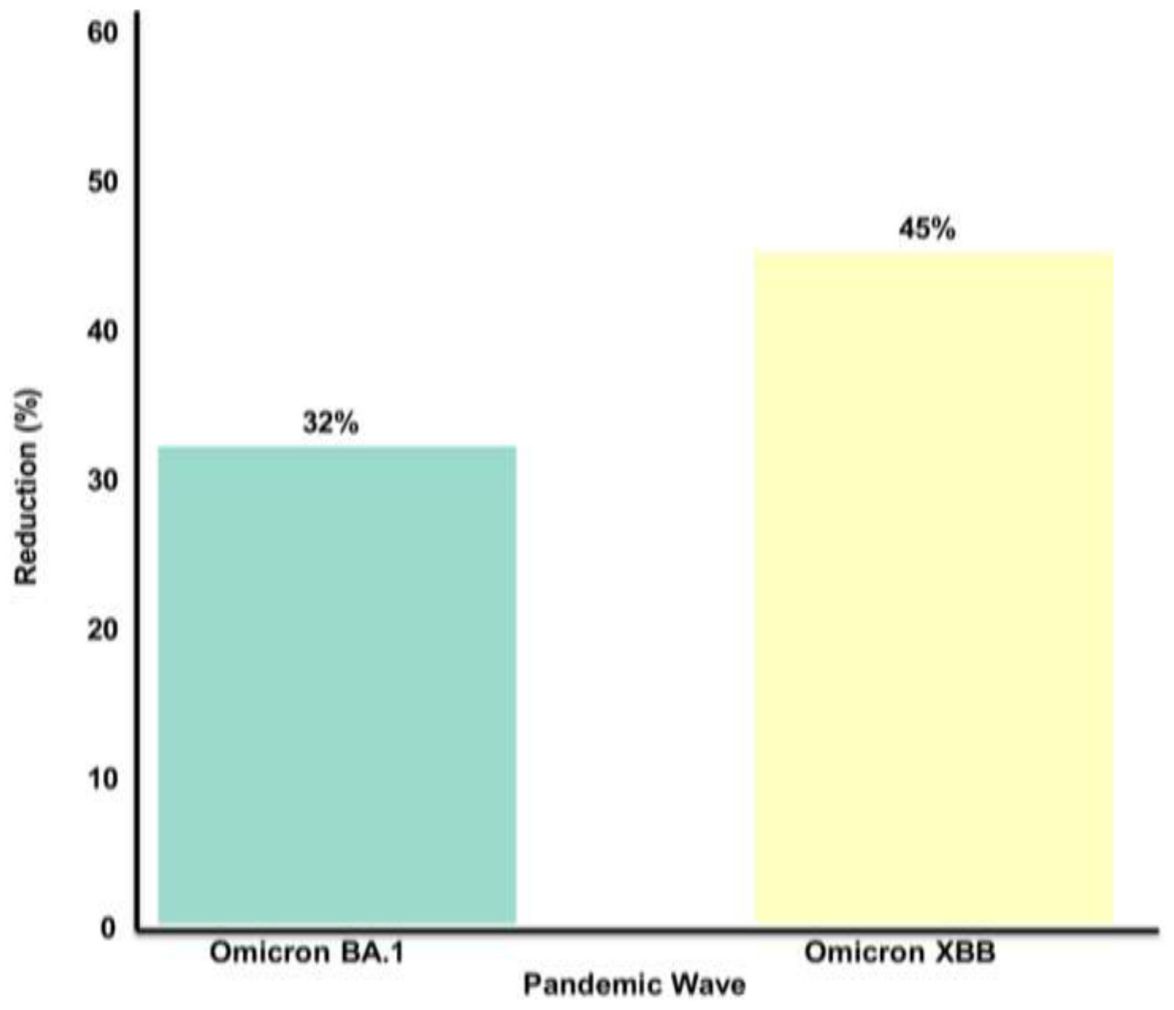
Vaccine-associated prevalence reduction in subsequent waves. The Omicron BA.1 wave showed a 32% reduction, and Omicron XBB demonstrated a 45% reduction compared with the pre-vaccine baselines. Comparative analysis of 42 studies with vaccination status data (*n* = 56,342 vaccinated patients).

Female predominance persisted across all waves but gradually attenuated. Vaccination was associated with substantial prevalence reductions of 32% during Omicron BA.1 and 42% during Omicron XBB compared to pre-vaccine baselines (56, 76, 77) (Figure 5).

## 4 Discussion

### 4.1 South Asia vs. global burden: a regional comparison

**Figure 6:**
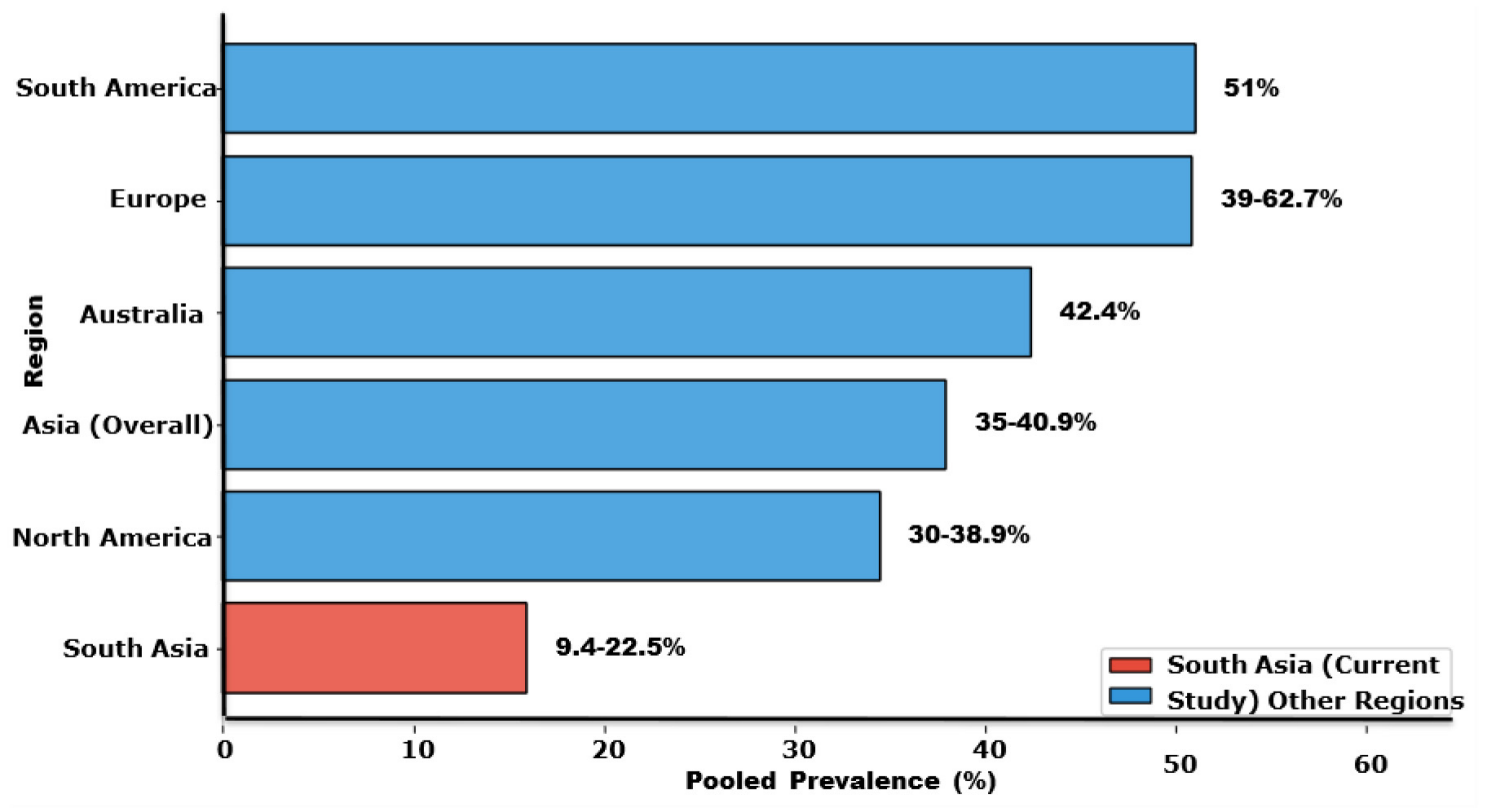
Overall post-COVID syndrome prevalence across South Asian countries. Pakistan had the highest pooled prevalence, followed by India and Bangladesh, based on 388 studies (7,237 initial records).

The findings of this review position the post-COVID syndrome burden in South Asia within the global context. While the pooled regional prevalence of 9.4%−22.5% under the WHO criteria appears moderate, this translates to 54.3 million affected individuals due to the region's large population base. Comparatively, South America has the highest global pooled prevalence at 51%, followed by Europe at 39%, Australia at 42.4%, Asia at 35%, and North America at 30% (10, 22). South Asia's apparently lower prevalence likely reflects substantial underdetection in rural areas, where limited diagnostic capabilities and healthcare-seeking behavior differences mask the true disease burden rather than indicating genuinely lower rates (8, 9, 23, 24, 48) (Table 4, Figure 6).

**Table 4:** Global Regional Comparison of Post-COVID Syndrome Prevalence.

Region	Pooled prevalence (%)	Key studies	Interpretation
South America	51%	Hou et al. (10, 48)	Highest global burden; broad definitions
Europe	39–62.7%	Razak et al. (22)	Higher in hospitalized populations
Australia	42.4%	Hou et al. (10, 48)	High detection, robust surveillance
Asia (Overall)	35–40.9%	Razak et al. (22)	Heterogeneous across subregions
North America	30–38.9%	Hou et al. (10, 48)	Lower; strong vaccination programs
South Asia	9.4–22.5%	Current review	Likely underdetected; 54.3M affected

### 4.2 Mechanistic interpretation of disparities

The consistent female predominance across all three countries (F:M ratios 1.41:1–1.81:1) suggests both biological susceptibility through estrogen-dependent immune responses, X-linked immune gene expression, and ACE2 receptor patterns, and social determinants, including caregiving roles and informal employment without health protections. This pattern mirrors global trends where female predominance transcends socioeconomic development levels (10, 78, 79). The peak burden in the 35–54 age group, corresponding to peak employment and family caregiving roles, carries profound economic implications for a region where this demographic constitutes the primary workforce.

Urban-rural disparities (Pakistan 3.1 pp, Bangladesh 6.1 pp, India 11.8 pp) correlated more strongly with healthcare infrastructure metrics than with infection severity. India's 11.8 percentage point gap the largest in the region reflects 80% concentration of diagnostic facilities in urban centers, higher urban healthcare utilization rates (3–4 × rural), and potential rural survivor bias where higher acute COVID-19 mortality may select for milder post-COVID syndrome cases (8, 9, 23, 24, 48). Bangladesh's divisional variations, with Khulna at 51.6% vs. Sylhet at 1.7%, further underscore the localized transmission dynamics shaping the burden distribution (12, 19).

### 4.3 Clinical implications

The multisystem nature of post-COVID syndrome, with fatigue (58.3%), brain fog (52.1%), and dyspnea (42.3%) as the leading manifestations, necessitates multidisciplinary management approaches. The discordance between subjective dyspnea (42.3%) and objective lung function decline (28.4%) indicates that rehabilitation must address both pulmonary and psychological components including anxiety, deconditioning, and sensory hyperawareness (54, 59, 80). Structured rehabilitation programs demonstrated significant improvement in 67.3% of patients, with telehealth approaches achieving 84% adherence and 40%−50% cost reduction, offering a viable model for rural healthcare delivery (81–85).

### 4.4 Healthcare system challenges

South Asian healthcare systems face critical capacity gaps: primary care facilities serve one per 30,000–50,000 population in rural areas vs. one per 5,000–10,000 in urban areas; diagnostic capabilities including cardiac imaging, pulmonary function testing, and cognitive assessment are available in only 40%−60% of urban vs. less than 5% of rural facilities; and rehabilitation services address only 2%−5% of identified needs (27, 59, 75, 86, 87). These infrastructure limitations both underestimate the true rural prevalence and prevent the adequate management of identified cases.

### 4.5 Implications for policy and future research

The declining prevalence across pandemic waves (32.1%−14.2%), coupled with vaccination-associated reductions (32%−42%), suggests that ongoing vaccination campaigns remain essential. However, the persistent burden of 14.2% even during Omicron XBB indicates that post-COVID syndrome will remain a significant healthcare challenge, requiring sustained investment. Future research priorities include South Asian-specific genetic studies (currently only 12 published), prospective cohorts with follow-up exceeding 18 months, and standardized WHO-criteria prevalence studies to enable accurate cross-regional comparisons (52, 88–92).

### 4.6 Practical recommendations

Dedicated post-COVID syndrome clinics need to be established within existing tertiary care centers in major cities across Pakistan, India, and Bangladesh, staffed by multidisciplinary teams, including pulmonologists, cardiologists, neurologists, psychiatrists, and rehabilitation specialists, with standardized WHO-criteria diagnostic protocols. Primary care physician training programs should be developed and implemented through online continuing medical education modules focused on post-COVID syndrome recognition, initial management, and referral criteria, targeting the primary care workforce as the first point of contact for most affected individuals. Rehabilitation infrastructure should be expanded by integrating post-COVID syndrome rehabilitation services into existing community health centers, leveraging telehealth platforms to extend specialist access to rural populations where only 2%−5% of rehabilitation needs are currently met. Support for vulnerable populations, particularly women aged 35–54 in informal employment who demonstrate the highest post-COVID syndrome burden, through workplace accommodations, targeted health screenings, and social protection programs, needs to be prioritized. Investment should be made in South Asian population-specific genetic research examining ACE2 polymorphisms, HLA variants, and interleukin gene profiles to identify region-specific susceptibility markers that can inform personalized therapeutic approaches. Regional surveillance systems should be strengthened by implementing standardized post-COVID syndrome registries across all three countries using WHO diagnostic criteria, enabling accurate prevalence monitoring, resource allocation planning, and evaluation of intervention effectiveness.

### 4.7 Strength

This review represents the first systematic synthesis integrating all major South Asian countries encompassing 2.1 billion population with diverse healthcare contexts (18, 24, 75, 93). The large evidence base of 388 peer-reviewed publications spanning 5.8 years and encompassing over 1.2 million participants, with GRADE quality assessment, enabled the prioritization of robust findings. Sex-specific, age-stratified, urban-rural, and temporal stratifications identify specific vulnerable populations requiring targeted interventions (74, 86, 94). PRISMA-ScR framework compliance with 87.3% inter-rater reliability at screening and 94.1% data extraction concordance ensures methodological rigor. Analysis of definition inconsistency explaining approximately 60% of prevalence variation provides mechanistic understanding, and implementation-focused recommendations include precise resource requirements and feasibility assessments (87, 95–97).

### 4.8 Limitations

Only 12 peer-reviewed studies have examined post-COVID syndrome genetic factors in South Asian populations despite 54.3 million affected individuals (52, 88–92). Pre-2023 studies employed variable case definitions contributing to approximately 60% of the 9.4%−34.2% variation; WHO 6-criterion definitions should be adopted in future research (10, 54, 98–101). Only 8.2% of the included studies provided follow-up data beyond 18 months, creating uncertainty regarding long-term disability trajectories. Vaccination during 2021–2025 complicates attribution of prevalence changes to viral variants vs. vaccination effects, although comparative studies suggest 40%−52% prevalence reduction with vaccination (56, 76, 77). English-language-only inclusion potentially excludes 5%−10% of relevant South Asian research, and indirect economic costs remain substantially underestimated (15, 24, 102–108).

## 5 Conclusion

Post-COVID syndrome poses a substantial and ongoing public health challenge in South Asia, affecting tens of millions of individuals across Pakistan, India, and Bangladesh and placing considerable strain on healthcare systems that are already operating under significant resource constraints. This systematic narrative review, synthesizing evidence from hundreds of peer-reviewed publications, reveals consistent patterns of female predominance and concentration of burden among working-age adults across diverse healthcare contexts, suggesting that biological vulnerability factors and gender-based socioeconomic determinants operate independently of healthcare development.

Urban-rural disparities in reported prevalence correlate strongly with healthcare infrastructure metrics rather than true epidemiological differences, indicating that the burden in underserved rural populations remains substantially under-detected and inadequately managed. The multisystem nature of post-COVID syndrome, with fatigue, cognitive impairment, and respiratory dysfunction as the leading manifestations, necessitates integrated multidisciplinary management approaches that extend beyond conventional single-system care pathways.

The declining prevalence across successive pandemic waves and the protective effect of vaccination provide a basis for cautious optimism. However, the persistent burden even during later variant periods underscores that post-COVID syndrome will remain a significant healthcare challenge, requiring sustained investment, research, and policy attention. Essential priorities include region-specific genetic research, long-term outcome studies, rehabilitation infrastructure development, and the establishment of standardized surveillance systems. With targeted healthcare system modifications and focused research on pathophysiology and outcomes, South Asia can reduce the post-COVID syndrome burden, enable functional recovery for affected populations, and advance the broader understanding of pandemic-associated chronic illnesses.
