# Supplementary material for: Post-COVID syndrome in Pakistan, India, and Bangladesh: a systematic narrative review of epidemiology, clinical manifestations, and healthcare system responses
Source: Front Public Health. 2026 May 20;14:1714880. doi: 10.3389/fpubh.2026.1714880 (PMC13230156; doi:10.3389/fpubh.2026.1714880)
Supplement: Supplementary file 1 [file Supplementary_file_1.docx]

**Additional File**

**Complete Database Search Strategies**

*Post-COVID Syndrome in South Asia: A Systematic Narrative Review*

**Overview**

This file provides the complete Boolean search strings used for each database in the systematic narrative review of post-COVID syndrome across Pakistan, India, and Bangladesh. Searches were conducted on November 19, 2025, covering the period January 2020- December 2023. Three databases were searched: PubMed, Scopus, and Google Scholar. All searches were restricted to English-language, peer-reviewed publications. Full details of the inclusion/exclusion criteria are provided in the main manuscript (Section 2.4).

**Database 1: PubMed**

**Search date: November 19, 2025**

Records retrieved: 3,847

*Interface: https://pubmed.ncbi.nlm.nih.gov/*

**Full Search String**

("Post-acute COVID-19 Syndrome"[MeSH Terms]
 OR "Long COVID"[tiab]
 OR "post-COVID syndrome"[tiab]
 OR "post-COVID condition"[tiab]
 OR "PASC"[tiab]
 OR "long-haul COVID"[tiab]
 OR "post-acute sequelae of SARS-CoV-2"[tiab]
 OR "post-acute COVID"[tiab])
AND
("South Asia"[tiab]
 OR "Pakistan"[tiab]
 OR "India"[tiab]
 OR "Bangladesh"[tiab]
 OR "Nepal"[tiab]
 OR "Sri Lanka"[tiab])
AND
("prevalence"[tiab]
 OR "epidemiology"[MeSH Terms]
 OR "clinical manifestations"[tiab]
 OR "risk factors"[MeSH Terms]
 OR "management"[tiab]
 OR "rehabilitation"[tiab]
 OR "fatigue"[tiab]
 OR "brain fog"[tiab]
 OR "dyspnea"[tiab])

Filters applied:
 - Publication date: 2020/01/01 to 2023/12/31
 - Language: English
 - Article types: Journal Article, Review, Systematic Review, Meta-Analysis

MeSH terms used: 'Post-acute COVID-19 Syndrome', 'Epidemiology', 'Risk Factors'. Free-text terms were applied in [tiab] (title and abstract) fields to maximise sensitivity. Geographic identifiers were not restricted to MeSH to capture varied terminology used in South Asian publications.

**Database 2: Scopus**

**Search date: November 19, 2025**

Records retrieved: 2,134

*Interface: https://www.scopus.com/*

**Full Search String**

TITLE-ABS-KEY(
 ("post-COVID syndrome" OR "long COVID" OR "post-acute COVID-19"
 OR "post-acute sequelae" OR "PASC" OR "long-haul COVID"
 OR "post-COVID condition" OR "post-COVID sequelae")
 AND
 ("Pakistan" OR "India" OR "Bangladesh"
 OR "South Asia" OR "Nepal" OR "Sri Lanka")
 AND
 ("prevalence" OR "epidemiology" OR "clinical manifestations"
 OR "risk factors" OR "management" OR "rehabilitation"
 OR "fatigue" OR "brain fog" OR "dyspnea" OR "gender"
 OR "urban" OR "rural" OR "pandemic wave")
)
AND PUBYEAR > 2019 AND PUBYEAR < 2024
AND DOCTYPE(ar OR re)
AND LANGUAGE(English)
AND SRCTYPE(j)

Scopus TITLE-ABS-KEY field searches titles, abstracts and keywords simultaneously. DOCTYPE(ar OR re) restricts to original articles and reviews. SRCTYPE(j) limits to journal sources, excluding conference proceedings and book chapters.

**Database 3: Google Scholar**

**Search date: November 19, 2025**

Records retrieved: 1,256

*Interface: https://scholar.google.com/*

**Full Search String**

"post-COVID syndrome" OR "long COVID" OR "PASC"
AND
("Pakistan" OR "India" OR "Bangladesh" OR "South Asia"
 OR "Nepal" OR "Sri Lanka")
AND
("prevalence" OR "epidemiology" OR "clinical manifestations"
 OR "risk factors" OR "rehabilitation")

Custom date range: January 2020 – December 2023
Language: English
Retrieval method: First 200 results per search query retrieved by
 relevance ranking, consistent with established methodology for
 LMIC-focused systematic reviews.
Total unique records screened from Google Scholar: 1,256

Google Scholar does not support MeSH indexing or structured Boolean operators in the same manner as PubMed and Scopus. It was included to improve sensitivity for regional grey literature, preprints, and South Asian journals not indexed in PubMed or Scopus, consistent with PRISMA-ScR guidance on comprehensive search strategies for scoping reviews.

**Deduplication and Record Management**

| **Stage** | **Records (n)** |
| --- | --- |
| PubMed | 3,847 |
| Scopus | 2,134 |
| Google Scholar | 1,256 |
| Combined initial yield | 7,237 |
| Duplicates removed (EndNote X20) | 1,425 |
| Unique records for screening | 5,812 |
| Duplicate removal accuracy (QC audit) | 98% |

**Notes**

- Search strings were developed iteratively by two reviewers and pilot-tested before the formal search date.
- Geographic identifiers included both country names and the regional term 'South Asia' to maximise recall.
- Temporal restriction (January 2020 - December 2023) was applied to align with the COVID-19 pandemic period.
- CINAHL, Embase, and Web of Science were not searched; justification is provided in Section 2.2 of the main manuscript.
- This file corresponds to Supplementary File 2 referenced in the main manuscript.
